# Supplementary figures and images for: Principal component analysis of alpha-helix deformations in transmembrane proteins
Source: PLoS One. 2021 Sep 15;16(9):e0257318. doi: 10.1371/journal.pone.0257318 (PMC8443038; doi:10.1371/journal.pone.0257318)

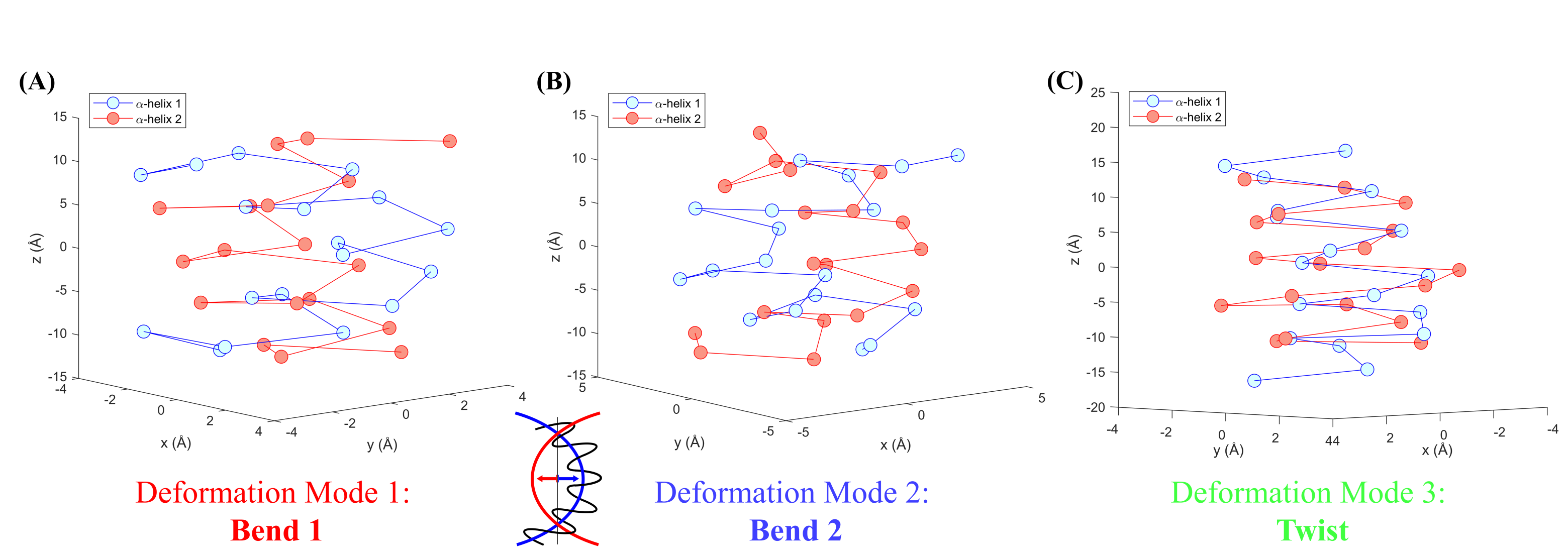

Supplement: S1 Fig — (A)-(C) In each subfigure, α-helix 1 and α-helix 2 are individual helices from the PDB in the transmembrane α-helix dataset. More specifically, they represent the two extreme cases of each deformation mode in the transmembrane α-helix dataset. (TIF) [file pone.0257318.s001.tif]

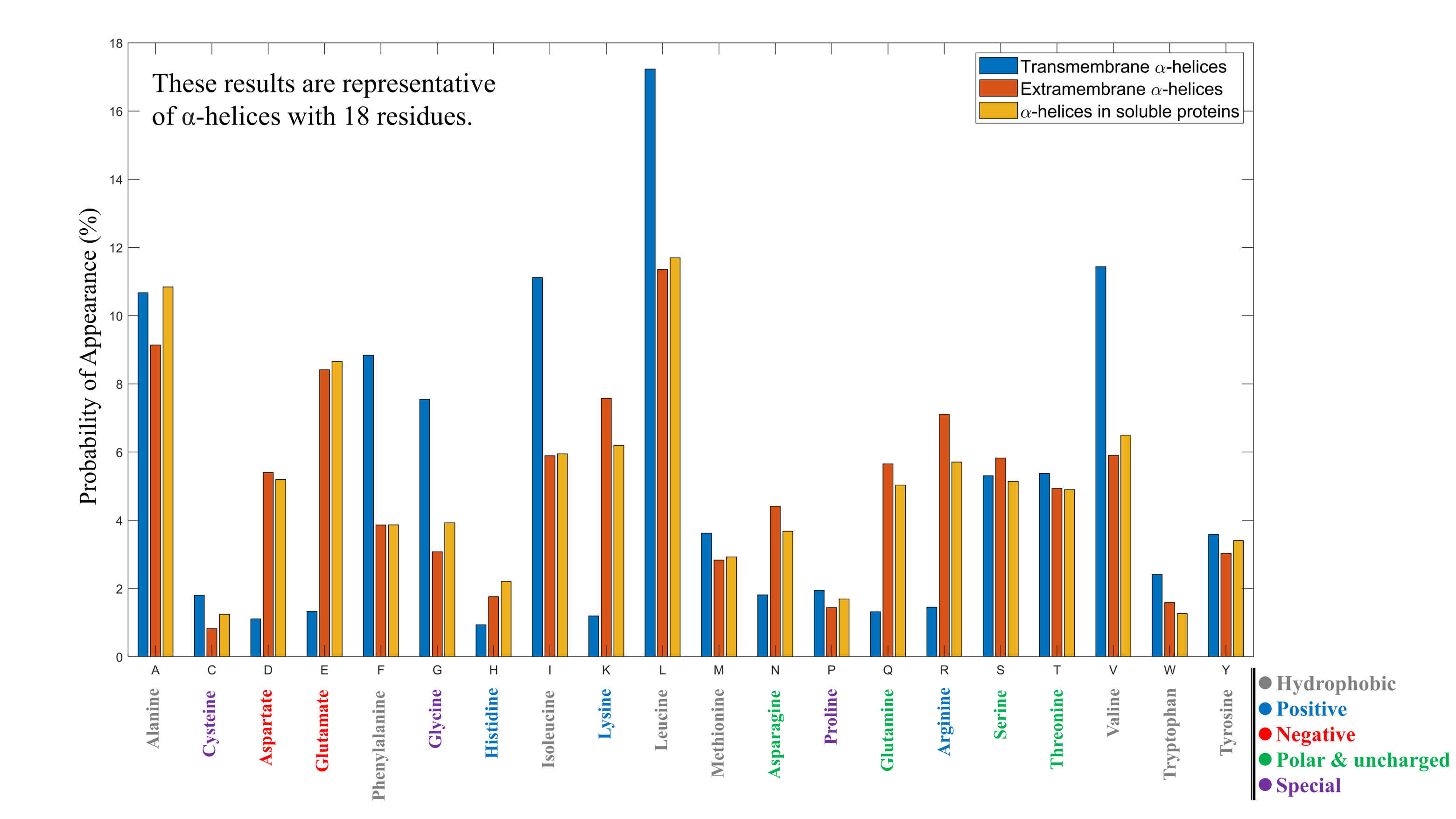

Supplement: S2 Fig — (TIF) [file pone.0257318.s002.tif]

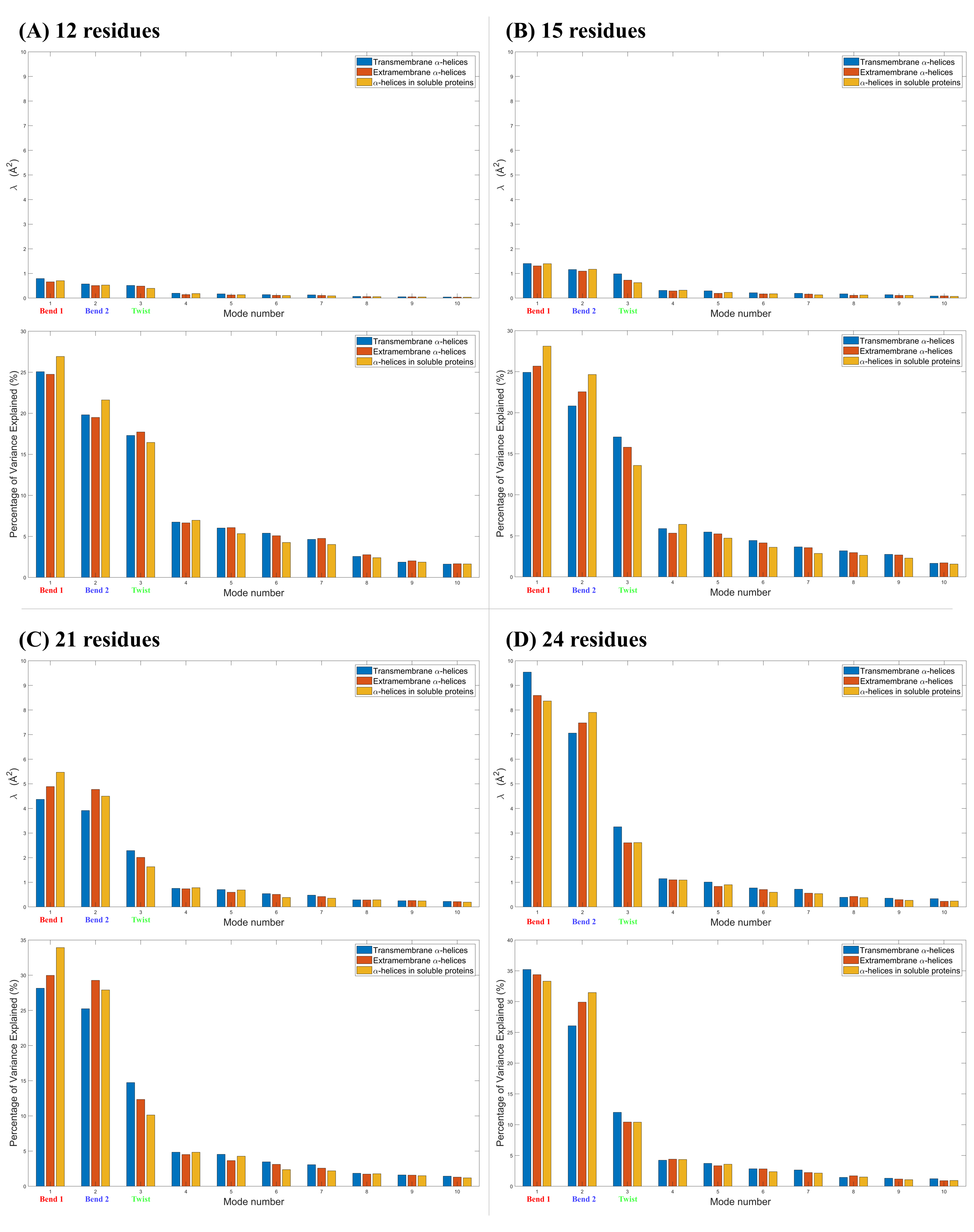

Supplement: S3 Fig — (TIF) [file pone.0257318.s003.tif]

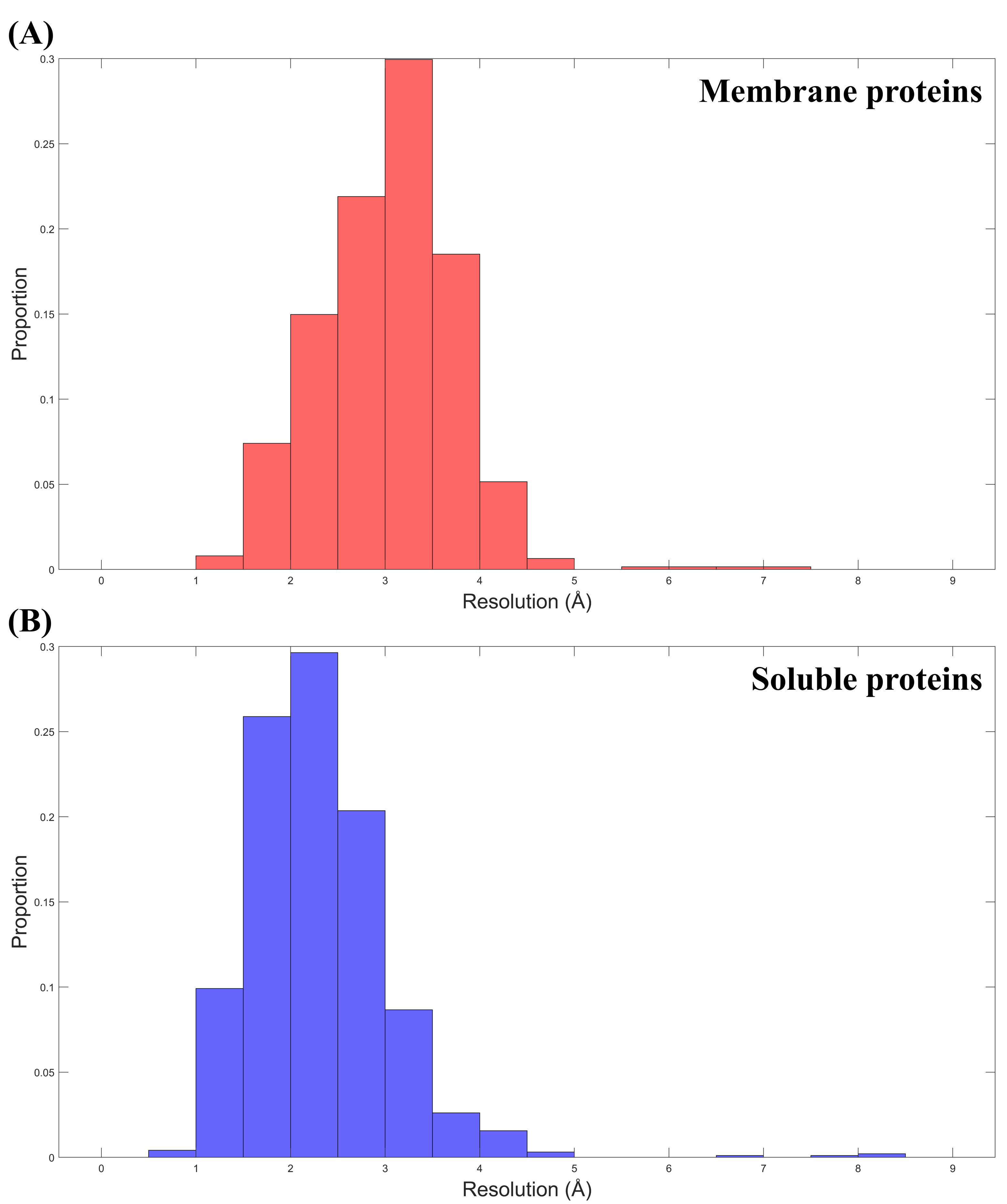

Supplement: S4 Fig — (A) The membrane protein PDB entries, specifically the transmembrane α-helix and extramembrane α-helix datasets, have an average resolution of 3.02 Å. (B) The soluble protein PDB entries used in this analysis have an average resolution of 2.31 Å. (TIF) [file pone.0257318.s004.tif]

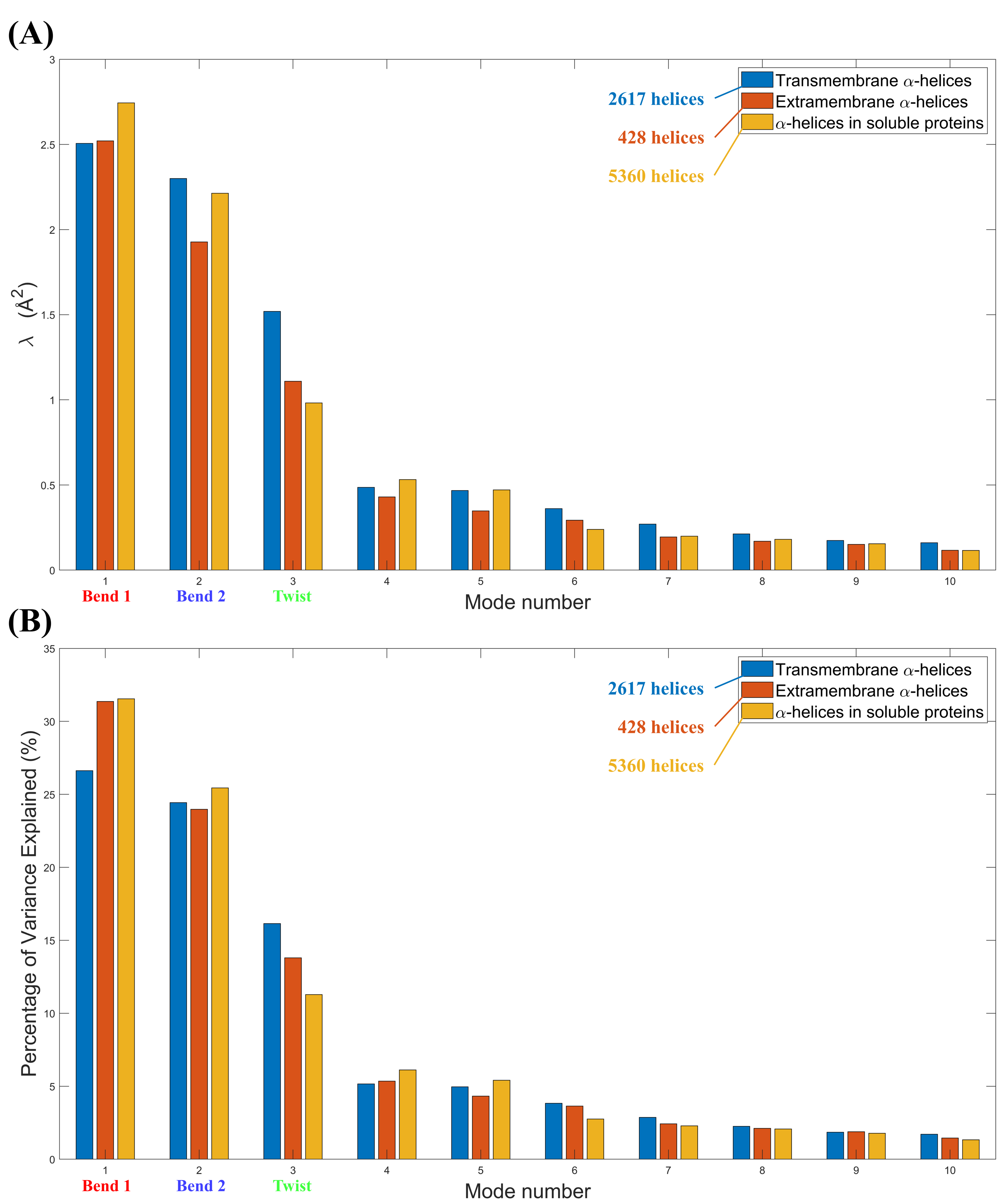

Supplement: S5 Fig — (A) The eigenvalues (λ). (B) The eigenvalues, when normalized by total variance. (TIF) [file pone.0257318.s005.tif]

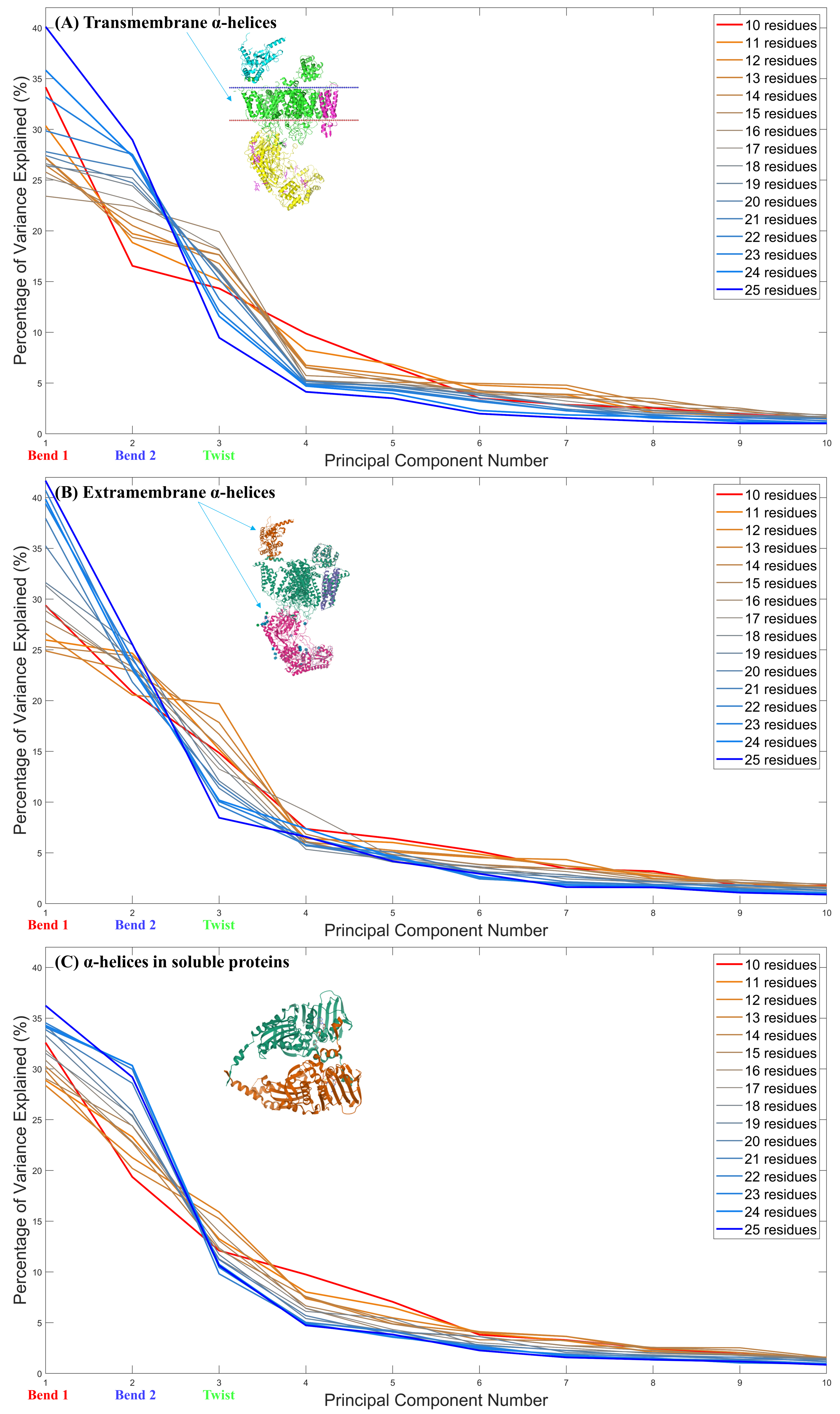

Supplement: S6 Fig — Sixteen lines are plotted to illustrate this trend in the range 10≤L≤25. The length of the α-helix in question is represented by the colour and thickness of each line. These distributions were plotted for (A) transmembrane α-helices, (B) extramembrane α-helices, and (C) α-helices in soluble proteins. (TIF) [file pone.0257318.s006.tif]

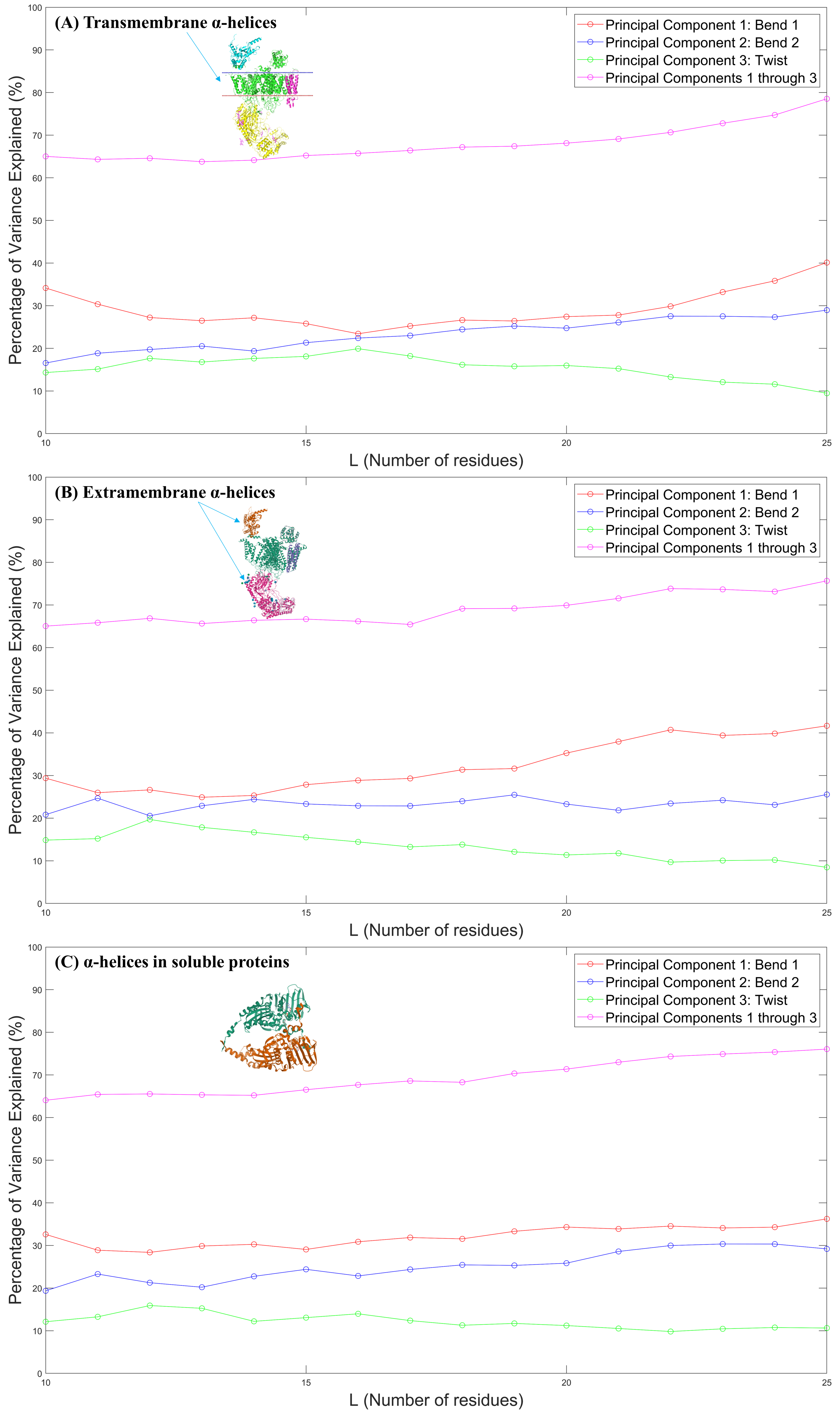

Supplement: S7 Fig — The percentage of total variance explained by each of the first three principal components individually (red, blue, and green) and combined (pink) for α-helices with helix lengths (L) in the range 10≤L≤25 for our analysis of only high−resolution structures (≤ 3 Å). The red, blue, and green lines represent the contributions of Bend 1, Bend 2, and Twist modes respectively towards explaining the total variance. The pink line represents the summed contributions of the first three principal components towards explaining the total variance. These results are plotted for (A) transmembrane α-helices, (B) extramembrane α-helices, and (C) α-helices in soluble proteins. (TIF) [file pone.0257318.s007.tif]

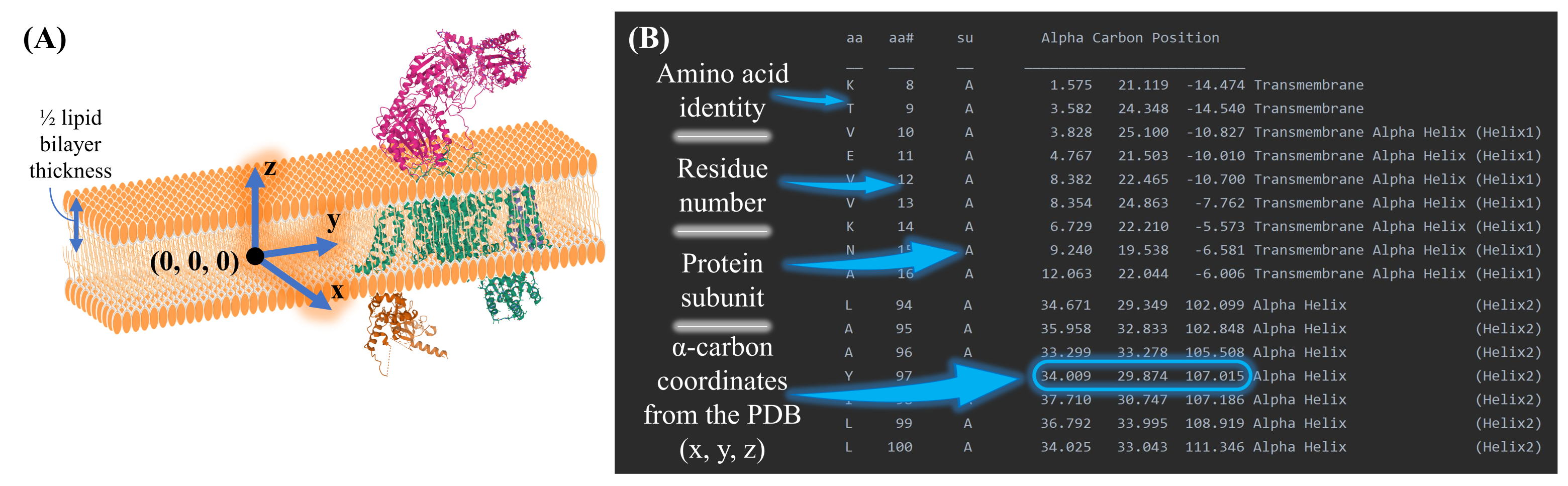

Supplement: S8 Fig — (A) A cartoon representation of transformed 3D atomic coordinates in the Orientations of Proteins in Membranes (OPM) Database. When the |zcoordinate|<½ lipid bilayer thickness, the α-carbon is part of a transmembrane region. (B) A piece of an outputted annotation text file: The preprocessed data from the RCSB and OPM PDB files include amino acid identity, residue number, protein subunit, α-carbon coordinates measured in Å, and the appropriate annotations. (TIF) [file pone.0257318.s008.tif]

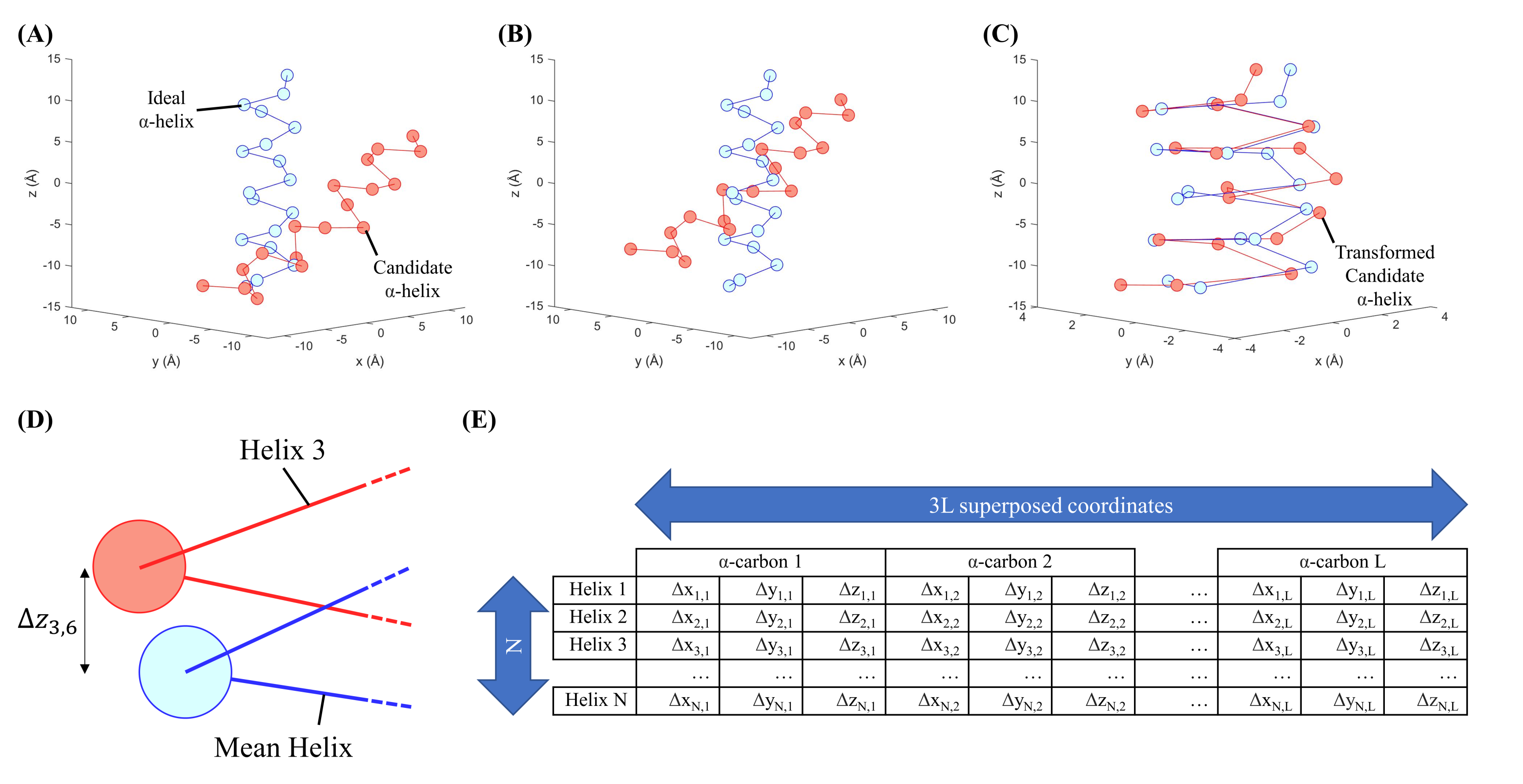

Supplement: S9 Fig — (A) The candidate α-helix and the ideal α-helix are not yet optimally superposed. (B) In the first step of superposition, the centroid of the candidate α-helix is translated to the origin. (C) In the second step of superposition, the candidate α-helix is rotated with respect to the ideal α-helix. (D) The displacement between the z-coordinate of α-carbon 6 in candidate α-helix 3 of the sample and the z-coordinate of α-carbon 6 in the mean α-helix is one of many data points in the raw data for PCA. (E) The raw data for PCA is an N by 3L matrix recording the displacements between each atomic coordinate of the transformed candidate α-helix and the corresponding atomic coordinate in the mean α-helix. (TIF) [file pone.0257318.s009.tif]
