# Supplementary material for: Principal component analysis of alpha-helix deformations in transmembrane proteins
Source: PLoS One. 2021 Sep 15;16(9):e0257318. doi: 10.1371/journal.pone.0257318 (PMC8443038; doi:10.1371/journal.pone.0257318)
Supplement: S1 Table — (DOCX) [file pone.0257318.s010.docx]

**S1 Table. The power law relationship between the eigenvalues (**$\boldsymbol{\lambda}$**) of the first three deformation modes and the α-helix length (**$\boldsymbol{L}$**).**

| $log\left( \lambda\right)=a\log\left( L \right)+b$ | | $a$ (slope) | $b$ (intercept) |
| --- | --- | --- | --- |
| **Bend 1** | Transmembrane α-helices | **3.27 (2.845, 3.695)** | -3.653 (-4.176, -3.129) |
|  | Extramembrane α-helices | **3.227 (3.031, 3.424)** | -3.578 (-3.82, -3.336) |
|  | α-helices in soluble proteins | **3.399 (3.18, 3.618)** | -3.786 (-4.056, -3.515) |
| **Bend 2** | Transmembrane α-helices | **3.597 (3.416, 3.778)** | -4.145 (-4.368, -3.922) |
|  | Extramembrane α-helices | **3.505 (3.385, 3.625)** | -3.97 (-4.119, -3.822) |
|  | α-helices in soluble proteins | **3.642 (3.453, 3.83)** | -4.175 (-4.407, -3.942) |
| **Twist** | Transmembrane α-helices | **2.725 (2.603, 2.848)** | -3.246 (-3.397, -3.095) |
|  | Extramembrane α-helices | **2.287 (2.087, 2.487)** | -2.739 (-2.986, -2.492) |
|  | α-helices in soluble proteins | **2.746 (2.617, 2.875)** | -3.395 (-3.554, -3.237) |

The power law relationship for transmembrane α-helices, extramembrane α-helices, and α-helices in soluble proteins was determined by establishing the best fit for the parameters $a$ and $b$ in $log\left( \lambda\right)=a\log\left( L \right)+b$. The scaling exponent (slope) and intercept are tabulated alongside their 95% confidence intervals in parentheses.
