# Supplementary material for: Principal component analysis of alpha-helix deformations in transmembrane proteins
Source: PLoS One. 2021 Sep 15;16(9):e0257318. doi: 10.1371/journal.pone.0257318 (PMC8443038; doi:10.1371/journal.pone.0257318)
Supplement: S2 Table — (DOCX) [file pone.0257318.s011.docx]

**S2 Table. The scaling exponents derived from a power law relationship between the eigenvalues (**$\boldsymbol{\lambda}$**) of the first three deformation modes and the α-helix length (**$\boldsymbol{L}$**) for our analysis of only high-resolution structures (**$\boldsymbol{\leq}$ **3 Å).**

| $\boldsymbol{\lambda\propto}\mathbf{L}^{\boldsymbol{∎}}$ | **Transmembrane α-helices** | **Extramembrane α-helices** | **α-helices in soluble proteins** |
| --- | --- | --- | --- |
| **Bend 1** | $\boldsymbol{3.2}$ | $\boldsymbol{3.3}$ | $\boldsymbol{3.2}$ |
| **Bend 2** | $\boldsymbol{3.6}$ | $\boldsymbol{2.9}$ | $\boldsymbol{3.4}$ |
| **Twist** | $\boldsymbol{2.7}$ | $\boldsymbol{2.1}$ | $\boldsymbol{2.6}$ |
